# Ketamine-assisted group psychotherapy integrating cognitive processing therapy to address identity-based trauma: a pilot study

**DOI:** 10.3389/fpsyt.2025.1727487

**Published:** 2026-01-22

**Authors:** Jae M. Sevelius, Rachel Lynn Golden, Brooke L. Stott, Natavi Orion Kozicz, Ronica Mukerjee, Sabrina R. Cluesman, Tennessee Jones, Talea Cornelius

**Affiliations:** 1 Department of Psychiatry, Columbia University Irving Medical Center, New York, NY, United States; 2 Golden Psychology, New York, NY, United States; 3 Columbia University, School of Social Work, New York, NY, United States; 4 Columbia University Irving Medical Center, School of Nursing, New York, NY, United States; 5 HIV Center for Clinical and Behavioral Studies, Division of Gender, Sexuality, and Health, Columbia University Irving Medical Center and the New York State Psychiatric Institute, New York, NY, United States; 6 Center for Drug Use and HIV Research, School of Global Public Health, New York University, New York, NY, United States; 7 Center for Behavioral Cardiovascular Health, Columbia University Irving Medical Center, New York, NY, United States

**Keywords:** cognitive processing therapy, gender minority, gender-expansive, group therapy, identity-based trauma, ketamine-assisted psychotherapy (KAP), psychedelic-assisted psychotherapy, transgender

## Abstract

**Introduction:**

Transgender and gender-expansive people experience high rates of depression, anxiety, and trauma-related distress, yet few evidence-based interventions are tailored to their needs. *Kindred* is a novel, group-based ketamine-assisted psychotherapy (KAP) program that integrates cognitive processing therapy (CPT) to address identity-based trauma.

**Methods:**

This mixed-methods pilot study evaluated the feasibility, acceptability, and preliminary effects of *Kindred* among eight transgender and gender-expansive adults. The nine-week group KAP intervention alternated ketamine dosing sessions with CPT-based cognitive skills-building and integration sessions in a community-based clinical setting. Quantitative measures assessed changes in mood, cognitive fusion, substance use, suicidality, trauma symptoms, and gender-related well-being from pre- to post-intervention, while qualitative interviews explored participants’ experiences and perceived mechanisms of change.

**Results:**

The intervention was feasible and highly acceptable, with 100% retention and high satisfaction ratings. Participants reported significant reductions in depression, anxiety, and cognitive fusion scores, alongside qualitative reports of decreased shame, suicidality, and internalized transphobia. Participants reported that group belonging, peer validation, and shared identity were important therapeutic factors that enhanced the impact of ketamine and CPT.

**Discussion:**

Findings suggest that *Kindred* is a feasible and promising intervention for addressing mental health symptoms of identity-based trauma among TGE adults. Integrating evidence-based psychotherapy, such as CPT, with KAP in a group setting may promote synergistic cognitive, emotional, and social mechanisms of healing while facilitating increased accessibility.

## Introduction

Transgender and gender-expansive (“trans”) people include individuals who are transgender, nonbinary, Two-Spirit, agender, or hold other gender identities that defy cisnormative expectations. Cisnormativity refers to the assumption that all people identify with the sex they were assigned at birth, and that such alignment is natural, expected, and socially privileged (1). Trans communities face extreme inequities in mental health compared to the general population, with disproportionately high rates of depression, anxiety, suicidality, and emotional dysregulation stemming from stigma and chronic traumatic invalidation (2). Chronic traumatic invalidation refers to the repeated dismissal, minimization, or rejection of a person’s thoughts, feelings, or experiences, often by those in positions of authority or within close relationships (3, 4). Among trans people, this can include having one’s identity dismissed and rejected, being misgendered repeatedly, and/or having one’s experiences of transphobia minimized by others (5, 6). These experiences are often compounded by intersecting oppressions including racism, heterosexism, and systemic cisnormativity, which shape how chronic invalidation is experienced across social contexts (7).

Over time, chronic experiences of traumatic invalidation can become internalized as rigid, global beliefs about oneself, others, and the world, particularly beliefs related to safety, trust, power, control, esteem, and intimacy (8). These inflexible cognitive patterns, often conceptualized as cognitive fusion or cognitive rigidity, limit the ability to evaluate thoughts flexibly or adaptively and are strongly associated with depression, anxiety, and trauma-related symptoms (9). Cognitive rigidity is increasingly recognized as a transdiagnostic process underlying diverse forms of psychopathology and treatment response (10). For trans and gender-expansive people, these rigid beliefs often reflect repeated exposure to chronic invalidation, making cognitive rigidity both a consequence of unmet need for gender affirmation and a barrier to recovery (11–13). Addressing these rigid, invalidation-based belief systems is therefore a critical therapeutic target for trans individuals experiencing chronic invalidation and identity-based trauma.

Psychedelic-assisted therapies (PAT), such as ketamine-assisted psychotherapy (KAP), offer new paradigms of mental health treatment by demonstrating safety and efficacy as catalysts for cognitive shifts that lead to long-term symptom relief and increased resilience (14).

Ketamine was approved by the US Food and Drug Administration in 1970 as an anesthetic; off-label use of ketamine, both alone and as an adjunct to psychotherapy, has been observed to improve symptoms of PTSD, depression, and other mood disorders in several studies (15–21). KAP currently offers several benefits over other types of PAT that use compounds such as psilocybin, MDMA, and LSD. Although psychotherapists themselves cannot prescribe, when clinical criteria are met, ketamine can be prescribed by a licensed medical provider and then legally self-administered by patients in sessions with psychotherapists, making it easier for psychotherapists to incorporate KAP into their current practices. On a federal level, other commonly used psychedelic compounds are currently Schedule 1 substances, making them illegal to access in most of the United States outside of clinical trials. Thus, legal access to these forms of PAT requires either costly travel to places where they are legal or enrollment in highly selective clinical trials that are expensive to implement, creating often insurmountable barriers to accessing urgently needed therapy and thereby widening disparities (22). Ketamine treatment also allows for flexible dosing and administration options, with shorter session durations than other commonly used psychedelic medicines (typically 2–4 hours), making it ideal for tailored therapeutic use (17, 23).

Despite the rapid growth of KAP, scholars in psychedelic studies have critiqued the field’s reliance on white, individualistic, and biomedical framings, which limit the field’s relevance and accessibility for marginalized communities (24). Very few KAP models have been developed by or for trans and gender-expansive people, and almost none explicitly address chronic invalidation and identity-based trauma. Approaches grounded in community, cultural attunement, and social determinants of health that incorporate intersectional frameworks responsive to LGBTQ+ lived experiences and needs, such as gender affirmation (25), are urgently needed (26–28). Further, research on the “social cure” highlights the powerful therapeutic potential of group contexts, which may be especially relevant in PAT (29, 30). For marginalized communities, group psychotherapy offers unique benefits: it reduces social isolation, provides opportunities for deep validation and shared understanding, and offers a safer environment with affirming role models (31). Delivering PAT in groups also reconnects the practice with its communal roots in traditional psychedelic use (24). Moreover, group-based KAP can lower implementation costs and thereby expand access for those with the greatest need (32). Together, these gaps point to a critical need for KAP interventions that integrate evidence-based psychotherapy with trauma-informed and community-centered approaches tailored for trans and gender-expansive people.

Researchers and therapists increasingly emphasize that PAT should be understood as a fully integrated treatment, rather than as drug administration accompanied by minimal or no psychological support (14, 33). In parallel, there is growing emphasis on selecting evidence-based psychotherapies to target specific psychological mechanisms to optimize treatment outcomes (23, 33, 34). In this study, we utilized cognitive processing therapy (CPT) (8), a cognitive behavioral therapy that has been used to treat PTSD, depression, anxiety and suicidality by helping individuals identify and modify rigid, trauma-related beliefs (35, 36). Because ketamine induces a temporary period of heightened neuroplasticity, it may facilitate cognitive restructuring during this window (37). Integrating CPT with ketamine may therefore support more effective examination and revision of rigid, trauma-based cognitions that arise from chronic invalidation and impact how people see themselves, others and the world. CPT teaches people to recognize maladaptive trauma-related beliefs and directly challenge them using cognitive skills that foster more flexible, balanced interpretations of their experiences. A combined CPT+KAP approach leverages emerging evidence that ketamine’s neuroplastic effects can increase psychological flexibility and openness to cognitive and emotional reframing, thereby facilitating the disruption of entrenched thought patterns and the acquisition of CPT skills (38, 39). By delivering CPT in close temporal proximity to ketamine dosing sessions, we sought to prepare participants for their KAP sessions and capitalize on the neuroplastic window to support challenging of beliefs, skills learning, and reappraisal of trauma-based cognitions, including internalized stigma and shame.

We developed a conceptual model informed by the hypothesis that the combination of ketamine’s pharmacological properties and CPT’s cognitive-behavioral strategies in a safe and affirming group context would promote synergistic improvements in mental health symptoms such as depression, anxiety, and suicidality by enhancing psychological flexibility and restoring a sense of agency among trans and gender-expansive individuals coping with chronic invalidation. To explore the benefits of this model, we developed an innovative intervention created by and for trans and gender-expansive people called “*Kindred*” to reflect the importance of community in healing and fostering a sense of safety.

The present pilot study had three primary aims. First, we sought to evaluate the feasibility and acceptability of *Kindred*; we expected high levels of retention, engagement, and satisfaction. Second, we aimed to explore preliminary changes in depression, anxiety, cognitive fusion, and other mental health outcomes from pre- to post-intervention; given prior evidence supporting both KAP and CPT, we expected decreases across these domains. Third, we examined exploratory associations between changes in hypothesized mechanisms (e.g., cognitive fusion and gender euphoria) and changes in mental health outcomes to generate hypotheses about potential pathways of therapeutic action. All outcome analyses were considered exploratory due to the small sample size and pilot design.

## Methods

### Study design

This pilot study employed a mixed methods convergent design (quantitative and qualitative) to examine the feasibility, acceptability, and preliminary effects of the *Kindred* intervention (40). The quantitative component utilized a single-group design (N = 8) with multiple assessment points: a baseline survey, a pre-intervention survey, a post-intervention survey, and repeated surveys after integration sessions (5 assessments) and self-administered medication sessions (4 assessments). The sample size was determined by feasibility and is consistent with pilot study designs (i.e., not powered for hypothesis testing). The qualitative component consisted of in-depth individual interviews conducted within a month of completing the 9-week trial, to explore participants’ perceptions of *Kindred*’s acceptability and feasibility and to collect feedback on the intervention design.

**Figure 1:**
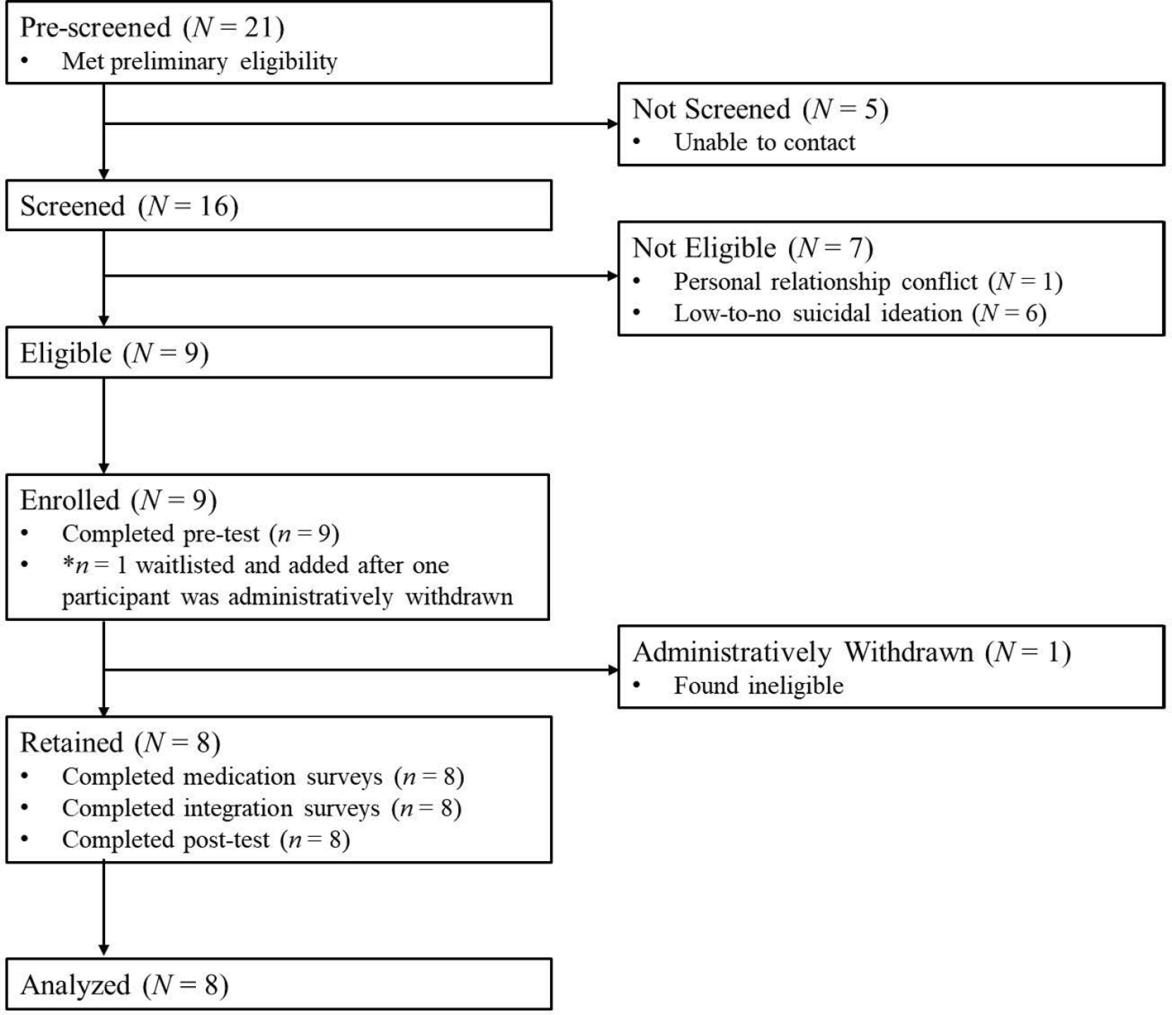
CONSORT diagram for Kindred study.

Consistent with a convergent mixed methods design, we collected quantitative outcome data and qualitative interview data in parallel to examine convergence and complementarity. These data sources were integrated during interpretation to assess how quantitative changes in symptoms and mechanisms aligned with or diverged from participants’ narratives of change and perceived therapeutic processes. Our study report follows the CONSORT extension for randomized pilot and feasibility trials with modifications to exclude randomization-related items (**Figure 1**) (41, 42).

### Ethical considerations

This study was designed in accordance with international ethical guidelines and was approved by the Columbia University Institutional Review Board. We emphasized in the consent process that participation in this study was voluntary and would have no impact on their mental health care, thus avoiding any undue pressure or coercion. All participants provided informed consent prior to baseline data collection. Participants received monetary compensation for their participation in the research as follows: $30 for completion of each of the 8 post-session surveys, $50 for a baseline survey before the first session, $50 for a post-intervention survey after the final session, and $50 for the completion of a qualitative interview conducted within the month following the final session. Total compensation for completing all study activities was $390. This study was funded by a grant from Healing Hearts, Changing Minds, Inc.; participation in the *Kindred* program was free to study participants.

### Setting and participants

*Kindred* was conducted in a community-based private psychotherapy practice in New York City. The practice is queer-owned and operated and dedicated to the affirming care of gender and sexuality expansive individuals and their families. Grounded in intersectional, justice-informed principles, they provide evidence-based therapies to address mental health challenges, including trauma, depression, anxiety, emotion dysregulation, suicidality and self-harm.

To be eligible for *Kindred*, participants were required to identify as transgender or gender-expansive, be 18 years of age or older, report gender dysphoria, report moderate to severe depression symptoms, have no dual relationships with study team (e.g., known to the study team in a social context, or be an existing client of the practice), be deemed suitable for ketamine administration after medical evaluation (see the following medication evaluation and dosing section for details), and be available to attend all sessions for the duration of the study. We recruited participants through digital study flyers that were circulated on relevant email lists, directing eligible people to apply to be screened for the study via a SurveyMonkey link. The link took applicants to a brief survey that served as an initial eligibility screening. Potentially eligible participants were then invited to an additional screening interview with the research assistant. Once deemed eligible, interested applicants were invited to schedule an enrollment appointment to review study procedures. At the enrollment appointment, study procedures were explained to the potential participant and informed consent was obtained by research staff.

### Medication evaluation and dosing

Each participant received a psychiatric evaluation to determine suitability for ketamine administration. Common medical and psychiatric contraindications were used to screen participant appropriateness, including recent (within 3 months) manic episode, significant history of psychosis, pregnancy, glaucoma, chronic ketamine overuse, and unstable cardiovascular disease. Participants were prescribed ketamine sublingual rapid dissolving tablets (RDTs) using weight-based dosing, with 5mg/kg as the starting dose for treatment as is typical of standardized KAP dosing protocols; 100 mg “boosters” of ketamine sublingual RDTs were also prescribed to augment dosing (43, 44). An ondansetron prescription, an anti-nausea medication, was also provided to participants to use as needed to mitigate the commonly experienced nausea effects of ketamine. Participants and therapists were encouraged to report back to the prescriber on ketamine effects and doses were increased or decreased as appropriate. Participants were instructed on how to appropriately prepare for the medication sessions, e.g., not eating immediately before sessions, not using substances or drinking alcohol the night before sessions, and how to appropriately self-administer the RDTs during the medication sessions, as study therapists would not be able to assist with this process. Participants were consistently and closely monitored by study therapists during dosing.

### Intervention design

We designed a 9-week intervention protocol by incorporating Cognitive Processing Therapy (CPT) into group KAP. CPT is a structured, evidence-based form of cognitive-behavioral therapy designed to help individuals process and recover from trauma (45). CPT focuses on identifying and challenging maladaptive thoughts, or “stuck points,” that arise from traumatic experiences and contribute to ongoing distress. Through guided discussions and structured exercises, individuals learn to recognize how their beliefs about themselves, others, and the world may have been altered by trauma and work to develop healthier, more adaptive cognitions (46).

**Figure 2:**
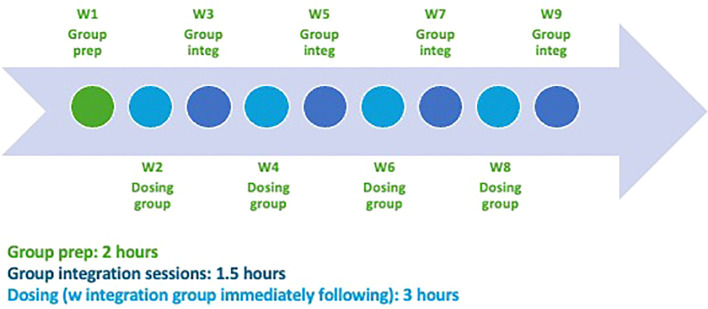
Kindred intervention structure.

In *Kindred*, CPT-based integration sessions were alternated with ketamine or “dosing” sessions over the 9-week intervention period (**Figure 2**). Participants engaged in an abridged individual intake to the therapy practice with one of the study therapists prior to the first group. All therapy sessions occurred in a group setting. Week 1 was a 2-hour group preparation session, which included an introduction to the group, other group members, the study therapists, group norms, KAP, and to the condensed skills-based CPT model, including psychoeducation about PTSD, related rigidity in thinking (“stuck points”), as well as preparation for the dosing sessions, providing time for group members to ask questions about the ketamine experience. Therapists also covered the type of therapeutic touch that could be provided upon request (e.g., briefly holding a hand, or touching a shoulder for support), and established guidelines for when participants were allowed to travel home and requiring that they be picked up by a chaperone. Therapists reinforced that they would not handle the medication, nor would they give advice about medication dosing for the duration of the study.

Dosing sessions were 3-hour ketamine sessions that followed a typical protocol of a grounding exercise and brief group meeting before the session, self-dosing ketamine experiential, and then a group integration after the session. During integration sessions, participants engaged in 1.5-hour group sessions to reflect and expand upon insights gleaned from the ketamine dosing sessions, during which they were also taught new CPT skills focused on addressing “stuck points” and negative thought patterns. Participants completed homework between sessions and received four half-hour, one-on-one coaching calls to support completion of the homework (e.g., developing a trauma narrative and identifying stuck points, using worksheets to practice and apply the weeks’ teachings to their own stuck points, and promoting engagement with the material). By addressing issues related to safety, trust, power, control, esteem, and intimacy, the goal of the CPT-based integration sessions was to help group members capitalize on the neuroplastic window opened by the ketamine to better uptake skills presented and challenge existing thought patterns during a time of increased cognitive flexibility, thereby gaining greater agency over trauma-based thought patterns. See **Table 1** for details related to the incorporation of CPT content into the group integration sessions.

**Table 1:** Outline of Kindred sessions and integration of CPT content.

Weeks	Session type	Incorporation of CPT content
Week 1	Preparation and introductions	Introduction of stuck points and impact statements
Week 2	Dosing	Review of stuck points
Week 3	Integration	Review of stuck points and impact statements, teach ABC worksheet
Week 4	Dosing	Review of impact of trauma on thoughts, feelings, and behaviors
Week 5	Integration	Review ABC worksheet, teach challenging questions worksheet
Week 6	Dosing	Explore willingness to challenge thoughts and change thinking
Week 7	Integration	Review challenging questions homework, teach patterns of problematic thinking worksheet
Week 8	Dosing	Explore willingness to change thinking patterns
Week 9	Integration	Review patterns of problematic thinking homework, teach and practice comprehensive worksheet

### Study therapists

The study therapists included one PhD clinical psychologist and two Licensed Master Social Workers (LMSWs) who provided support during groups; the same therapists facilitated all sessions except for one dosing session covered by an alternate therapist known to the group. One of the study therapists conducted all coaching calls between group sessions. The other therapists also provided brief check ins if needed with participants between group sessions. All therapists identified as transgender or gender expansive and have notable experience working clinically within these communities. All therapists had formal training specific to the provision of ketamine-assisted psychotherapy and experience with individual ketamine-assisted psychotherapy. Two therapists had prior experience with group-based ketamine-assisted psychotherapy, comprehensive trainings and certificates in psychedelic-assisted therapy and research, and additional training in psychedelic integration.

### Data collection

Enrolled participants provided a preferred cell phone number or email address to which links to study surveys were sent. Prior to the initial group session, participants were sent a link to the baseline survey, which was programmed in Qualtrics. Subsequently, participants received links to surveys after each group session and a final post-test survey after completion of the final intervention session. Within one month following all group sessions and the post-test survey, the research staff conducted a one-hour qualitative interview with each participant. Data collection occurred between May and September 2024.

### Measures

#### Feasibility and acceptability

Feasibility measures included rates of recruitment and retention of clients, amount and type of resources needed for implementation, and any adjustments that should be made for future implementations. We additionally gathered self-report data from participants in the post-intervention survey using the Feasibility of Intervention Measure (FIM), the Acceptability of Intervention Measure (AIM), and the Intervention Appropriateness Measure (IAM) (47). Each of these 4-item Likert scales are scored from 1, “Completely disagree,” to 5, “Completely agree.” A total score is computed for each scale by taking a mean of the items, with a scale score of 4 (“Agree”) or greater indicating agreement that the intervention was feasible, acceptable, or appropriate. We also measured program satisfaction on a scale of 1 (very dissatisfied) to 4 (very satisfied) with three questions that asked participants to rate the quality of the therapy they received, to what extent their needs were met, and how satisfied they were at the end of each *Kindred* session.

#### Preliminary effects

Quantitative measures included the following outcomes of interest:

##### Patient health questionnaire-8

The PHQ-8 is an 8-item self-report measure designed to assess the severity of depressive symptoms based on DSM-IV criteria (48). It omits the item on suicidal ideation found in the PHQ-9. Respondents rate the frequency of symptoms over the past two weeks on a 4-point Likert scale ranging from 0 (not at all) to 3 (nearly every day), yielding a total score from 0 to 24, with higher scores indicating more severe depression.

##### Generalized anxiety disorder-7

The GAD-7 is a 7-item self-report questionnaire developed to assess the severity of generalized anxiety symptoms over the past two weeks (49). Each item corresponds to DSM-IV criteria for generalized anxiety disorder and is rated on a 4-point Likert scale ranging from 0 (not at all) to 3 (nearly every day), yielding a total score from 0 to 21. Higher scores reflect greater anxiety severity. The GAD-7 has demonstrated strong reliability, construct validity, and sensitivity to change, and is widely used in both clinical and research settings.

##### Trauma symptoms due to gender invalidation (adapted from the PCL-5)

The PTSD Checklist for DSM-5 (PCL-5) is a 20-item self-report measure designed to assess symptoms of posttraumatic stress disorder (PTSD) as defined by the DSM-5 (50). The PCL-5 can be used for screening, diagnosis, and monitoring symptom change, and has demonstrated strong psychometric properties across diverse populations. We adapted the measure to assess trauma symptoms due to gender invalidation by revising the instructions as follows: “*Below is a list of problems that people sometimes have in response to experiences of having their gender invalidated by other people. For example, experiences of gender invalidation might include being misgendered, such as someone using the wrong pronoun for you, having someone address you by a former name, or feeling stigmatized or discriminated against because of your gender identity or expression*.” We also adapted the response options such that the term “invalidation” was used instead of the original phrase “stressful experience”. Respondents rated how much they have been bothered by each symptom in the past month on a 5-point Likert scale ranging from 0 (not at all) to 4 (extremely), yielding a total severity score from 0 to 80.

##### Columbia-suicide severity rating scale

The C-SSRS is a structured clinician-administered or self-report instrument designed to assess the severity and intensity of suicidal ideation and behavior (51). We used the 6-item version to capture a range of ideation (from passive thoughts to active suicidal intent with a plan), as well as preparatory acts, actual attempts, and non-suicidal self-injury. The scale has demonstrated strong predictive validity and is widely used in clinical and research settings to evaluate suicide risk.

##### Drug abuse screening test–10

The DAST-10 is a 10-item self-report screening instrument designed to identify potential problems related to drug use (excluding alcohol and tobacco) over the past 12 months (52). Each item is answered with a yes/no response, and total scores range from 0 to 10, with higher scores indicating greater levels of drug-related problems. The DAST-10 is a widely used, psychometrically validated tool in clinical and research settings for quickly assessing the severity of drug misuse.

#### Hypothesized intervention mechanisms

##### Cognitive fusion questionnaire–7 (CFQ-7)

The CFQ-7 is a 7-item self-report scale developed to measure cognitive fusion, a core process in psychological inflexibility where individuals become entangled with their thoughts (53). Respondents rate each item on a 7-point Likert scale ranging from 1 (never true) to 7 (always true), with higher scores indicating greater cognitive fusion. The CFQ-7 has demonstrated strong internal consistency and construct validity and is commonly used in studies involving Acceptance and Commitment Therapy (ACT) and related psychotherapy models.

##### Difficulties in emotion regulation scale (DERS)

The DERS is a 36-item self-report scale developed to assess multiple aspects of emotion dysregulation (54). It includes six subscales: nonacceptance of emotional responses, difficulties engaging in goal-directed behavior, impulse control difficulties, lack of emotional awareness, limited access to emotion regulation strategies, and lack of emotional clarity. Participants rate items on a 5-point Likert scale ranging from 1 (almost never) to 5 (almost always), with higher scores indicating greater difficulties in emotion regulation.

##### Mystical experiences questionnaire (MEQ)

The MEQ is a 30-item self-report scale developed to measure the phenomenological features of mystical-type experiences occasioned by psychedelic substances (55, 56). Items assess domains such as unity, transcendence of time and space, ineffability, and sacredness. Each item is rated on a 6-point Likert scale ranging from 0 (not at all) to 5 (extreme; more than ever before). The MEQ has been widely validated in psychedelic research and is sensitive to changes in intensity and quality of altered states of consciousness. The MEQ has demonstrated excellent internal consistency (α ≈ 0.90) and strong construct validity across clinical, experimental, and naturalistic contexts.

##### Gender euphoria

Gender euphoria is a positive feeling associated with one’s gender identity, expression, or affirmation. To assess gender euphoria, participants responded to a six-item measure using the response options “Disagree”, “Agree”, “Strongly agree”, or “Prefer not to answer”. The item stem was “When I think about my gender identity or gender expression, I feel a deep sense of:” and the terms included words indicating positive emotions such as “contentment”, “satisfaction”, and “joy”.

##### Gender minority stress and resilience

The GMSR scale is a self-report measure a validated 58-item measure developed to capture both stressors and resilience processes unique to transgender and gender-expansive populations (57). The scale assesses nine constructs: three distal stressors (gender-related discrimination, gender-related rejection, gender-related victimization); three proximal stressors (non-affirmation of gender identity, internalized transphobia, negative expectations for future events); and three resilience factors (including community connectedness, and pride). Items are rated on either 4 or 5-point Likert scales, with higher scores on stressor subscales indicating greater exposure to minority stress, while higher scores on resilience subscales indicate stronger adaptive resources.

#### Qualitative interviews

We documented experiences of Kindred among participants by conducting individual in-depth qualitative interviews via Zoom (N = 8) post-implementation to explore clients’ overall experience of the group, including perceived impact of the group on their mental health, and to solicit feedback to improve future implementation. A research assistant (NO) conducted the interviews, each of which lasted approximately 60 minutes.

### Data analyses

#### Sample size determination

Sample size was guided by the study aim of informing feasibility, acceptability, and appropriateness of Kindred for improving mental health symptoms of identity-based trauma among TGE adults (N = 8). Thus, quantitative data on preliminary effects are exploratory and descriptive only, as this study is not powered to detect significant changes from pre- to post- *Kindred* implementation.

#### Quantitative analyses

For variables described using means and standard deviations, changes from pre-intervention to post-intervention were tested using paired t-tests, and Cohen’s d was calculated as the mean of the pre-post change divided by the standard deviation of change. For variables described using medians and interquartile range, nonparametric Wilcoxon signed rank tests were used, and effect sizes were reported as a correlation, calculated as the test statistic divided by the square root of the sample size. We also estimate and describe correlations between study variables. Correlations among change scores were exploratory and not corrected for multiple comparisons, consistent with the pilot nature of the study.

#### Qualitative analyses

Interviews were recorded via Zoom. After the interview, the research team deleted the video recording and saved the audio recording to an encrypted shared drive for transcription. Audio recordings were deleted once the transcript was finalized. After interviews were transcribed verbatim and de-identified, the transcripts were imported into Dedoose, a qualitative coding software program. A team of 4 coders (SRC, ER, AM, NO) independently coded each transcript using a codebook developed by the study team, with oversight by a senior qualitative researcher (JS). Any differences in code application were resolved during interactive discussions. No major discrepancies occurred, indicating high coder agreement. We used a deductive, thematic analysis approach informed by our conceptual model, such that initial codes reflected hypothesized mechanisms of change and key domains of participant experience (including feasibility and acceptability of the intervention), while allowing space to capture unanticipated themes. Quotes are identified using pseudonyms; only general information is provided about participants’ race and age by decade to protect participants’ identities.

## Results

In May 2024, we pre-screened 21 potential participants. Of those, five people never responded to screening attempts and 16 people were screened. Seven people screened ineligible and 9 people screened eligible (see CONSORT diagram for details). Of these, 9 were enrolled and one person was waitlisted due to further medical testing required before medical clearance could be granted. After enrollment, one participant was deemed ineligible due to inability to attend all sessions and was withdrawn by the study team. After this participant was withdrawn, the waitlisted participant was enrolled following medical clearance, resulting in 9 enrolled total, 8 eligible and 8 retained.

Mean age of participants was 27 years (SD 3.38); half (n=4, 50%) self-identified as a person of color. The sample was relatively well-educated, with the majority reporting some college or a college degree (n=6, 75%); the remaining had an advanced or professional degree (n=2, 25%). Diverse genders and sexual orientations were represented; half (n=4, 50%) were nonbinary and more than a third were trans femme/trans women (n=3, 37.5%), and the majority identified as queer and/or pansexual (n=5, 62.5%, for both identities). Most had extensive prior experience with psychotherapy (n=7, 87.5%) but none had prior experience with therapeutic ketamine (n=0). See **Table 1** for additional demographic data.

### Feasibility and acceptability

All participants (100%) attended all intervention sessions. All 8 completed both the pre-test and post-test surveys. The four medication surveys also had a 100% completion rate amongst the 8 participants; one participant declined to respond to communality items during the third survey and one participant declined to respond to gender euphoria items during the fifth survey. The five integration surveys also had 100% completion rates. The participant whose enrollment was delayed did not respond to the preparation session survey because they missed the group session, so their preparation session was conducted individually.

Participant ratings of intervention feasibility, acceptability, and appropriateness were high (**Table 2**), indicating participants felt the intervention was feasible, acceptable and appropriate to implement in their communities. Satisfaction with the intervention was also high.

**Table 2:** Demographics for *N* = 8 participants.

Variable	Participant Characteristics	Mean (SD) or N(%)
Age		27.63 (3.38)
Race/Ethnicity*	Asian	1 (12.5%)
	Black	1 (12.5%)
	Latine	2 (25%)
	Indigenous/First People	1 (12.5%)
	Middle Eastern/N. African	3 (37.5%)
	Mixed	1 (12.5%)
	White	4 (50%)
Identification as Person of Color	Yes	4 (50%)
	No	4 (50%)
Education	Some College	3 (37.5%)
	College Degree	3 (37.5%)
	Advanced/Professional Degree	2 (25%)
Income Source*	Full-time Employment	5 (62.5%)
	Part-time Employment	3 (37.5%)
	Financial support from family	2 (25%)
	Student income (e.g., loans)	1 (12.5%)
	Unemployment	1 (12.5%)
Living Arrangement	Owns housing	1 (12.5%)
	Rents housing	6 (75%)
	Lives with family	1 (12.5%)
Gender*	Nonbinary	4 (50%)
	Trans	1 (12.5%)
	Nonbinary Trans	2 (25%)
	Transfemme/Trans woman	3 (37.5%)
	Gender Nonconforming	1 (12.5%)
Sexual Orientation*	Pansexual	5 (62.5%)
	Queer	5 (62.5%)
	Bisexual	1 (12.5%)
	Lesbian	1 (12.5%)
Relationship status	Single	1 (12.5%)
	Dating (Casual Relationship)	3 (37.5%)
	Committed Relationship	3 (37.5%)
	Nonhierarchical/Poly	1 (12.5%)
Prior experience with psychotherapy	0 times	1 (12.5%)
	1-20 times	0 (0.0%)
	More than 20 times	7 (87.5%)
Prior experience with recreational ketamine	0 times	6 (66.7%)
	1-10 times	2 (25%)
	10-20 times	0 (0.0%)
	More than 20 times	1 (12.5%)
Prior experience with therapeutic ketamine	None	8 (100.0%)

*Note that some categories sum to greater than 8, as participants were able to select multiple options.

### Preliminary effect on mental health outcomes

**Figure 3:**
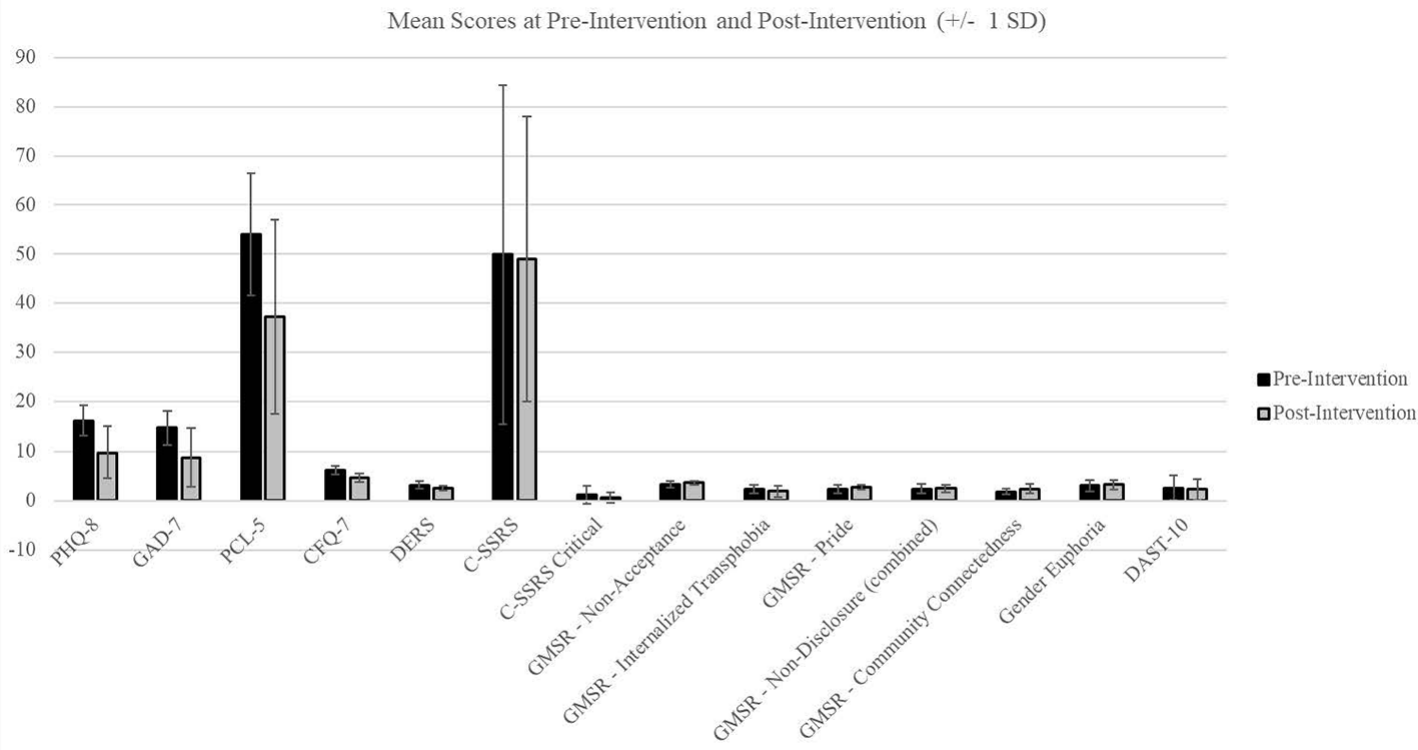
Mean scores at pre-intervention and post-intervention (+/- 1 SD).

We observed significant pre-post reductions on two primary outcomes of interest (depression, anxiety), as well as significant improvement on one of the hypothesized mechanisms (cognitive fusion). There was a significant decrease in depression scores from pre- to post-intervention (M = 16.13 (SD = 3.04), M = 9.75 (SD = 5.20), p=.019; *Cohen’s d* = 1.08), as well as a significant decrease in anxiety scores from pre- to post-intervention (M = 14.63 (SD = 3.34), M = 8.75 (SD = 5.90) p=.030; *Cohen’s d* = 0.96). We also found a significant decrease in cognitive fusion scores (M = 6.20 (SD = 0.91), M = 4.66 (SD = 0.94), p=.002; *Cohen’s d* = 1.78). Changes in other mental health outcomes and mechanisms of interest (e.g., emotional dysregulation, suicidality, trauma symptoms) were not significant in this small sample but changes noted were in the expected directions (**Tables 2**, **3**) (**Figure 3**). To further explore potential mechanisms of change, we examined correlations between hypothesized mediators (e.g., cognitive fusion, gender euphoria) and changes in mental health outcomes (**Table 4**). Notably, reductions in cognitive fusion were strongly associated with decreases in depression, anxiety, and PTSD symptoms.

**Table 3:** Psychosocial variables at pre-test and post-test (N=8).

Measure	Mean (SD) or Median [IQR]		*Cohen’s d* or *r*	*p*-value
	Pre-test	Post-test		
PHQ-8 Sum Score	16.13 (3.04)	9.75 (5.20)	1.08	.019*
GAD-7 Sum Score	14.63 (3.34)	8.75 (5.90)	0.96	.030*
DERS Mean Score	3.18 (0.70)	2.57 (0.51)	0.78	.064
PCL Sum Score	54.00 (12.40)	37.25 (19.75)	0.77	.065
*Additional non-parametric test*	54.0 [17.0]	45.5 [38.5]	0.77	.078
Cognitive Fusion Mean Score	6.20 (0.91)	4.66 (0.94)	1.78	.002**
CSSRS Sum Score	49.88 (34.49)	49.00 (29.08)	0.04	.90
*Additional non-parametric test*	43.0 [62]	47.5 [50.5]	0.04	.84
CSSRS Critical Items	0 [2]	0 [1]	0.25	.63
Gender Minority Stress and Resilience – Non-Acceptance Mean Score	3.35 (0.65)	3.63 (0.34)	0.62	.12
Gender Minority Stress and Resilience – Internalized Transphobia Mean Score	2.36 (0.92)	1.91 (1.19)	0.66	.10
Gender Minority Stress and Resilience – Pride Mean Score	2.34 (0.80)	2.69 (0.51)	0.48	.21
Gender Minority Stress and Resilience – Non-Disclosure Mean Score	2.43 (1.04)	2.45 (0.71)	0.03	.94
Gender Minority Stress and Resilience – Non-Disclosure (does not live as affirmed gender almost/all of the time) Mean Score	2.75 (0.87) *n = 4	2.80 (0.60) *n = 3		
Gender Minority Stress and Resilience – Non-Disclosure (lives as affirmed gender almost/all of the time) Mean Score	2.10 (1.23) *n = 4	2.24 (0.74) *n = 5		
Gender Minority Stress and Resilience – Community Connectedness Mean Score	1.78 (0.57)	2.38 (0.97)	0.62	.13
Gender Euphoria Mean Score	3.04 (1.15)	3.25 (0.98)	0.32	.40
DAST Sum Score	3 [4]	1.5 [3.5]	0.35	.50
Satisfaction		3.83 (0.27)		
Intervention Acceptability Mean Score		4.81 (0.29)		
Intervention Appropriateness Mean Score		4.78 (0.36)		
Intervention Feasibility Mean Score		4.38 (0.67)		

For statistics reported as mean and standard deviation, pared t-tests were used and Cohen’s d calculated. For statistics reported as median and interquartile range, Wilcoxon signed rank tests were used and r calculated. For acceptability, appropriateness, and feasibility, possible scale range was 1—5. In the mean/median columns, * is used to indicate that the n analyzed is different for this variable than for the overall sample. in the p-value column, * is used to flag a p-value less than .05, and ** is used to flag a p-value less than .01.

**Table 4:** Correlations between change in hypothesized mechanisms and change in clinical outcomes from pre-test to post-test.

Mechanism	Depression	Anxiety	PTSD symptoms	DERS (Difficulties in emotion regulation)	ASIQ (Suicidality)	ASIQ critical items	DAST (Substance use)
Cognitive Fusion	.81*	.83*	.94**	.51	.48	.34	.11
Gender Euphoria	–.74*	–.74*	–.74*	–.28	–.51	–.40	–.11
GMSR (Gender Identity)	.33	.29	–.01	.01	.40	.18	.33
Group Cohesion (last)	–.24	.00	–.56	–.08	–.38	–.51	.48
Group Cohesion (mean)	–.33	–.11	–.56	–.37	–.44	–.46	.23

Values represent Pearson correlations between change scores (pre–post). *p < .05, **p < .001.

### Qualitative findings

To complement the quantitative results, we conducted qualitative interviews to capture participants’ lived experiences of Kindred. The qualitative findings deepen our understanding of feasibility and acceptability, highlight mechanisms of change not fully captured by quantitative measures, and illuminate opportunities for improving the structure and delivery of the intervention. We report here on themes that represent the most common and robust findings across participants.

#### Reflections on the group setting

Participants emphasized the importance of the group context, describing it as central to the intervention’s impact. They described how the group normalized diverse expressions of transness, reduced shame, and fostered lasting connection. These findings also include mutual support and role modeling as key therapeutic factors. One participant described how experiencing ketamine-assisted therapy in a group setting with other trans and gender expansive people impacted her sense of self.

*“I am in such a better place with my gender identity than I was before … being around trans and gender expansive people [was] a reminder that transness can look so many different ways … as a trans feminine person, there’s so much pressure to be the most passable woman ever, it was really helpful to be reminded: Yeah, that’s bullshit. That really, really helped me.” (Ellis, 20s, White)*

For many participants, the group context offered opportunities to reflect on and gain insight about one’s own experiences by listening to the experiences of others. As one person shared, being in the group was like looking into a mirror; it helped them develop self-compassion by seeing parts of themselves in their peers.

*“Hearing from other people in real time was really profound. It was like an opportunity to have a lot of reflections, like mirrors, which gave me the opportunity to find compassion for myself by listening to another person who was saying things that I have felt. I went into this thinking, “I don’t know about this whole group thing,” and now I’m like, “Oh yeah, let’s do a group thing.” (Zephyr, 30s, mixed race).*

Sage reflected on the value of the group for working with the CPT skills, the ongoing impact of the program on their life, and the need for making this work more widely available:

*“It’s hard to imagine what the trajectory of my life would have been without [the Kindred program]. When I was saying [the CPT exercise] is only helpful because everyone in the group is here, and if I’m doing it by myself, it’s not very helpful. [The facilitator] said, you could imagine you can take everyone with you in your heart and imagine they’re there - that was so sweet. So, I’m carrying it around, the whole experience, in how I navigate my life, and my gender, and my well-being. It was such an impactful experience … I hope this becomes more available because ketamine is already so inaccessible and doing it in this kind of setting is just so beautiful.” (Sage, 30s, White)*

Participants also offered practical recommendations to enhance both the depth and continuity of care. Some participants described wanting to get to know the other group members better prior to the first ketamine dosing session. They described bonding with the other group members as one of their favorite parts of the program, as Sage went on to describe:

*“I do wish there was a little bit more of a chance to know the other group members before we did the ketamine … I think we got there eventually anyway … [before sessions] we would chat and that was one of my favorite parts. When I started to hear more of their stories coming out in the group, it did make me feel even closer to them. So I think it could have been cool to get more of a session where we shared more of our backgrounds a little bit, or why we were there early on.” (Sage, 30s, White).*

#### Structure and duration of sessions

Participants reflected on the structure and pacing of the program, noting areas where adjustments could enhance both therapeutic depth and integration. Several emphasized the need for scheduled breaks during integration sessions and more time during dosing sessions, particularly given individual differences in how participants metabolized ketamine.

*“The integration sessions were generally a good [length of] time, but I think we needed a break in between … I have a hard time sitting still for two hours. For the medicine ones, it was almost too short. I think I had a slower metabolism and I felt like I didn’t have enough time or like I was missing stuff.” (Auden, 20s, Mixed race)*

*“We came up right against the end of the sessions, a lot … an extra half hour on those dosing sessions would be helpful. (Ren, 30s, White).*

Many felt that in addition to longer sessions, extending the overall program duration and building in dedicated reflection time between meetings would better support the gradual processing of insights and integration into daily life.

*“We always kind of ran out of time, like we’d get around the circle and then there would be five minutes for the last person to talk. Sometimes people didn’t want to, but in my opinion that shouldn’t be because of time … I consistently felt like I wished we had just a little more time for this.” (Zephyr, 30s, mixed race)*

While regular check-ins with a therapist were scheduled between sessions to review CPT homework and skills, some participants wanted additional scheduled check-ins after dosing sessions to prevent or address short-term destabilization.

*“After the three ketamine sessions where I had the full dose, it would have been really nice to have a little check in because I was having the experience of a very nice ketamine session, and then I would just completely crash after, and I would be so in the depths, and very dysregulated. That might have been a nice opportunity to practice some of the skills, or even just check in. I was fine. And I guess I could have reached out, but I didn’t, because I didn’t want to bother anybody.” (Sage, White, 30s)*

While participants overwhelmingly reported satisfaction with the group sessions, many wanted more and longer sessions. These reflections echo group therapy literature suggesting that adequate time and containment are crucial for processing emotionally intense material. In psychedelic-assisted contexts, insufficient time for sharing may limit opportunities for collective meaning-making and integration. At the same time, extending session length or program duration must be balanced with keeping the intervention accessible: acknowledging the limits and costs of facilitators’ time, minimizing participant fatigue, and ensuring the overall commitment remains manageable and sustainable.

To sustain improvement and maintain connections post-program, participants recommended tangible scaffolds such as structured post-session check-ins, optional peer support channels, and mechanisms for ongoing contact once the group concludes. Several participants noted that creating intentional structures for ongoing connection could help preserve therapeutic gains, foster continued belonging, and reinforce the protective effects of community.

*“I really wish there was more intention around creating a way for us all to stay connected. We exchanged information, but we haven’t made a group chat or been in communication, and I’m personally missing that. If there was just a forum created for us, where people could choose to participate or not, it would take that burden off of us.” (Zephyr, 30s, mixed race)*

#### Mental health benefits

Participants described profound shifts in how they related to their thoughts and emotions since the completion of the *Kindred* intervention. One of the most notable findings was a significant reduction in anxiety, and increased flexibility in decision making for some, a behavioral outcome of improved cognitive flexibility.

*“My anxiety has been significantly better … I was going through a bad episodic thing, but now I feel better overall. A big thing I noticed was less decision paralysis. I used to be overwhelmed by every option—even simple things like, should I do laundry or go to the grocery store? That was huge … Now I don’t feel that way anymore, and the overall sense of doom and dread is a little bit gone.” (Ellis, White, 20s)*

Participants described pronounced anti-anxiety and anti-depressant effects from the ketamine sessions, reporting that their mood lifted noticeably in the days after each dosing session, as exemplified by Indigo’s account.

*“The benefits of the therapy were a lot more comprehensive than I expected … I got anti-anxiety and anti-depressant benefits that I think were purely from the ketamine … By the end, it was consistent: after a session, I’d have two days where I felt worse, then 12 days where everything was phenomenally easier and calmer, and then it would fade until the next session.” (Indigo, White, 20s)*

In addition to these pharmacological effects, participants reported that the group structure and CPT skills uptake supported lasting change, with some describing new automatic responses to entrenched thoughts. Indigo also reported reductions in OCD symptoms since completing the *Kindred* program. Unlike past attempts at CBT techniques that never stuck, they now found themselves able to challenge their OCD and anxiety with CPT strategies.

*“I’ve noticed long-lasting benefits for my OCD tendencies. We did a lot of CBT practices I’d done before, but for the first time ever, it was like things stuck. A week later, I’d notice my brain doing it on its own. Every few days I’d have some OCD or anxiety thought, and I could feel my brain reacting differently. That was the biggest thing I noticed, along with the anti-depressant and anxiety effects.” (Indigo, 20s White)*

Alongside this, participants reported marked reductions in suicidality and increases in positive experiences.

*“Thinking about where I started and where I am now … I’m able to do the work that used to matter to me. The first thought in my mind when I wake up isn’t about killing myself, and that doesn’t come up very often anymore. The anhedonia is gone … I’m noticing the small things again, the things I used to be grateful for … that is back.” (Sage, 30s, White)*

A few participants who had taken or were taking SSRIs or other psychiatric medications reported that the ketamine sessions provided unique therapeutic depth and insight beyond their existing regimens. For example, Sage describes how SSRIs hadn’t worked for them, while their participation in *Kindred* resulted in palpable symptom relief:

*“I tend to respond poorly to most psych meds in general, and I have responded really poorly to all the different antidepressants I tried. Yet, I would say that participating in this group has been very, very helpful for my depression and my anxiety is like night and day. Overall, I feel a lot less anxious.” (Sage, 30s, White)*

## Discussion

Findings from this pilot study suggest that *Kindred*, a CPT-informed, ketamine-assisted group psychotherapy (KAP) model tailored for transgender and gender-expansive individuals, is both feasible and acceptable. Participants demonstrated high engagement, with 100% session attendance throughout the intervention. Although quantitative results are preliminary and should be interpreted with caution, results were promising. We observed significant reductions in depression, anxiety, and cognitive fusion, while other outcomes changed in the expected direction. A central aim of this pilot was to employ a mixed methods design that would allow quantitative outcomes and qualitative narratives to inform one another. Our preliminary quantitative findings align closely with qualitative accounts in which participants described personally meaningful decreases in depression, anxiety, suicidality, and rumination, along with noted improvements in decision-making, mood, and overall well-being. Importantly, participants qualitatively described strengthened community ties and a renewed sense of agency, suggesting that *Kindred’s* effects may operate through both intrapersonal mechanisms (e.g., reduced attachment to rigid, trauma informed cognitions, improved positive emotions, and behavioral engagement in change) and interpersonal mechanisms (e.g., identity affirmation, social connectedness). These preliminary findings indicate potential effects on mental health outcomes that warrant further investigation in larger, randomized controlled trials powered to rigorously evaluate intervention efficacy.

One of the most notable quantitative findings from this study was the significant reduction in cognitive fusion, a core component of psychological flexibility characterized by rigid adherence to thoughts, including obsessive and ruminative thinking, difficulty considering alternative perspectives, heightened emotional reactivity, and related behavior. Cognitive fusion is particularly relevant for trans and gender expansive people, as chronic invalidation and internalized stigma can reinforce negative self-beliefs and perpetuate maladaptive cognitive patterns (58). The decrease in cognitive fusion observed both quantitively and qualitatively suggests that *Kindred’s* combination of CPT targeting cognitive rigidity paired with ketamine-induced increased neural plasticity may help participants relate to their thoughts more flexibly, allowing them to question internalized beliefs in a more adaptive and less distressing way.

Prior research suggests that ketamine’s unique psychopharmacological effects enhance psychological flexibility, a process strongly linked to improved mental health outcomes and reduced cognitive fusion (23). Ketamine produces rapid antidepressant effects by blocking NMDA receptors, leading to increased glutamate signaling, activation of AMPA receptors, and the growth of new synaptic connections (59, 60). These neurobiological changes are thought to restore flexibility and connectivity in brain circuits involved in mood regulation (61). In parallel, ketamine’s dissociative and psychedelic effects may facilitate shifts in perspective by allowing individuals to experience distance from entrenched thought patterns, processes that are related to but distinct from its biological mechanisms (60). Our findings are consistent with this literature, suggesting that ketamine may reduce cognitive fusion by creating transient states of dissociation or altered consciousness that allow participants to experience a sense of distance from previously entrenched self-narratives (37). Together, these effects may create an optimal window for cognitive restructuring, particularly when paired with structured psychotherapies such as CPT that provide explicit tools for identifying and reframing rigid trauma-based beliefs (62).

Emerging evidence supports the premise that combining ketamine with structured psychotherapy can enhance and extend clinical benefits, potentially reducing relapse compared to ketamine alone (18). Our findings align with this literature, as participants described not only short-term symptom relief but also enduring shifts in cognitive patterns, supporting the hypothesis that ketamine’s neuroplastic window may increase the effectiveness and durability of evidence-based interventions such as CPT. By teaching participants how to challenge maladaptive cognitive patterns and replace them with more adaptive beliefs, CPT also provided a clear framework for integrating the insights gained during dosing sessions. This highlights the importance of combining ketamine with evidence-based psychotherapy models, rather than relying on the pharmacological effects alone (23).

A key strength of *Kindred* lies in its implementation of KAP with CPT in a group setting. This design leverages the unique synergies created by combining these approaches, allowing participants to process trauma within a structured framework while also benefiting from the communal aspects of peer modeling and support. In the integration sessions, group members were able to observe as others challenged existing beliefs, and internalized new ones about themselves, others and the world. Modeling of cognitive transformation likely influenced other group members to challenge their own beliefs. In addition, prior research has shown that group therapy can be particularly beneficial for marginalized communities, as it provides opportunities for connection, validation, and identity affirmation (63). The qualitative data further reinforced this, with participants emphasizing the significance of shared experiences and unique sense of safety fostered by a trans-centered therapeutic space. While the mechanism of action for psilocybin is very different than that of ketamine, a pilot study on the effects of group-administered psilocybin on psychological flexibility found large reductions in cognitive fusion at both 2-week and 6-month follow-ups (64). It is possible that the group experience may also be a notable factor in decreasing cognitive fusion among participants as observing others gain flexibility with their thinking may provide a model for increasing flexibility in one’s own thinking.

Reducing cognitive fusion may be particularly beneficial for trans and gender expansive individuals struggling with internalized transphobia and self-stigmatization. Many participants in our study described shifts in how they viewed their gender identity, moving away from rigid, externally imposed narratives toward a more expansive and self-affirming perspective. This finding is consistent with recent research indicating that ketamine, as well as other psychedelics, can promote shifts in self-concept and enhance self-compassion, particularly when integrated into psychotherapy (65, 66). Future research should further explore the role of psychological flexibility as a potential mechanism underlying the therapeutic effects of KAP in trans and gender-expansive populations, as well as other communities that face social stigma and structural oppression.

Our findings support the use of ketamine in populations with high rates of psychiatric medication use. Unlike classical psychedelics such as MDMA or psilocybin, which often require discontinuation of SSRIs and other psychiatric medications due to potential adverse interactions, ketamine can be safely administered alongside these treatments (67, 68). Requiring individuals to discontinue existing psychiatric medications poses substantial risks, including the potential for withdrawal symptoms and increased suicidality. This is particularly critical for trans and gender expansive individuals, who report disproportionately high rates of psychiatric medication use (69). The ability to integrate ketamine into existing medication regimens enhances its accessibility and safety profile for this population. Furthermore, adverse events were monitored throughout dosing sessions via direct therapist observation and participant self-report. No serious adverse events occurred, which further contributes to safety information about ketamine administration as an adjunct to group therapy. This finding reflects broader reports that significant adverse effects from ketamine administration are rare (70).

Despite promising results, this study has several limitations. The sample size was small, as it was a pilot study designed to help us explore feasibility, acceptability, and preliminary effects. Thus, quantitative results can be considered exploratory and descriptive only. The study was conducted in a single community-based clinical setting in New York City; therefore, findings may not translate to other geographic or clinical contexts, particularly those in non-urban settings or outside of the United States. Lack of a control group prevents us from determining whether the observed changes were directly attributable to the *Kindred* intervention or other external factors. Although Kindred’s group-based, trans-affirming model may reduce some financial and cultural barriers, broader structural issues such as out-of-pocket cost of KAP, insurance coverage, provider availability and training in LGBTQ+ affirming care, and medical mistrust persist. Addressing these inequities will be essential as KAP models move toward broader dissemination.

Future research should seek to evaluate *Kindred* in a larger, randomized controlled trial to more rigorously assess its efficacy and mechanisms of action. In addition, future research should examine strategies for scaling models like Kindred while preserving core therapeutic elements. Hybrid delivery formats (e.g., in-person ketamine dosing combined with telehealth-based preparation and integration sessions) may reduce geographic and financial barriers and extend access for trans and gender-expansive individuals. In addition, training peer facilitators to support CPT-based integration under appropriate clinical supervision may enhance scalability while maintaining safety and fidelity. A growing body of research suggests that peer-based facilitation is a critical mechanism for effective intervention delivery in trans and gender-expansive communities, supporting engagement, trust, and sustained participation in mental health and behavioral health programs (71–73).

In conclusion, our pilot study provides preliminary evidence that *Kindred* is a feasible, acceptable, and potentially effective intervention for addressing identity-based trauma among trans and gender expansive individuals. The integration of KAP and CPT within a structured group therapy model represents a novel approach to addressing the profound mental health disparities faced by this population. As Bennett and colleagues note, ethical ketamine practice requires integration support before, during, and after administration (74). The *Kindred* model directly responds to this call by embedding ketamine within a structured group psychotherapy framework. Findings are preliminary but promising; further research is warranted to explore the scalability and long-term impacts of *Kindred* in diverse settings and with other marginalized communities.

## Data Availability

The datasets presented in this article are not readily available because the small number of participants makes them potentially identifiable. Requests to access the datasets should be directed to js6254@cumc.columbia.edu.
